# *MGMT*基因启动子甲基化与非小细胞肺癌关系的*meta*分析

**DOI:** 10.3779/j.issn.1009-3419.2014.08.04

**Published:** 2014-08-20

**Authors:** 念珍 房, 俊东 谷, 慧君 魏, 嘉琮 尤, 清华 周

**Affiliations:** 1 300052 天津，天津医科大学总医院，天津市肺癌研究所，天津市肺癌转移与肿瘤微环境重点实验 Tianjin Key Laboratory of Lung Cancer Metastasis and Tumor Microenvironment, Tianjin Lung Cancer Institute, Tianjin Medical University General Hospital, Tianjin 300052, China; 2 300121 天津，天津市人民医院胸外科 Department of Thoracic Surgery, Tianjin Union Medical Center, Tianjin 300121, China

**Keywords:** 肺肿瘤, *MGMT*基因, 甲基化, *meta*分析, Lung neoplsams, *MGMT* gene, Methylation, *Meta*-analysis

## Abstract

**背景与目的:**

抑癌基因启动子区域甲基化是基因失活的重要机制之一, 本研究采用*meta*分析的方法探讨非小细胞肺癌(non-small cell lung cancer, NSCLC)患者癌组织与自身对照组织(血浆、正常肺组织及支气管灌洗液)*MGMT*基因启动子甲基化率有无差别。

**方法:**

计算机检索Medline、EMBASE、CNKI及万方等数据库, 收集公开发表的涉及*MGMT*基因启动子甲基化与NSCLC关系的临床研究。采用*meta*分析的方法比较NSCLC患者癌组织与正常自身对照组织中*MGMT*基因启动子甲基化率有无差别。

**结果:**

15篇文献符合纳入标准并纳入本研究, NSCLC患者肺癌组织中*MGMT*基因启动子甲基化率为38%(95%CI:23%-53%); NSCLC患者正常肺组织、血浆和支气管灌洗液中*MGMT*基因启动子甲基化率分别为16%(95%CI:5%-27%)、23%(95%CI:10%-34%)和39%(95%CI:23%-55%)。与正常肺组织和血浆比较, 肺癌中*MGMT*基因启动子甲基化率增高(OR=3.98, 95%CI:2.71-5.84, *P* < 0.05)(OR=1.88, 95%CI:1.16-3.05, *P* < 0.05), 与支气管灌洗液比较差别无统计学意义(OR=2.05, 95%CI:0.88-4.78, *P* > 0.05)。

**结论:**

NSCLC患者肺癌组织中*MGMT*基因启动子甲基化率增高, 该基因的启动子甲基化与肺癌的发生可能存在相关性。

## Abstract (en)

**Backgroud and objective:**

DNA promoter methylation of the tumor suppressor genes was one of the key mechanism for gene silence.The aim of this study is to investigate the difference of *MGMT* gene promoter methylation rate in tumor tissue and autologous controls (serum, normal lung tissue and bronchial lavage fluid) in patients with non-small cell lung cancer (NSCLC).

**Methods:**

The databases of Medline, EMBSE, CNKI and Wanfang were searched for selection of published articles of *MGMT* gene promoter methylation and non-small cell lung carcinoma risk.The pooled odds ratio (OR) and percentage of MGMT for lung cancer tissue of NSCLC patients compared with normal lung tissue, plasma and the bronchial lavage fluid were pooled.

**Results:**

15 articles of association between *MGMT* gene promoter methylation and non small cell lung carcinoma risk were included in this meta-analysis.The combined results demonstrated the methylation rate of *MGMT* in NSCLC cancer tissue was 38% (95%CI:23%-53%).For normal lung tissue, plasma and the bronchial lavage fluid were 16% (95%CI:5%-27%), 23% (95%CI:10%-34%) and 39% (95%CI:23%-55%) respectively.The OR in cancer tissue was much higher than that in normal lung tissue and plasma odds ratio (OR) 3.98 (95%CI:2.71-5.84, *P* < 0.05) and OR 1.88 (95%CI:1.16-3.05, *P* < 0.05), but not in bronchial lavage fluid OR 2.05 (95%CI:0.88-4.78, *P* > 0.05).

**Conclusions:**

Mehtylation rate in *MGMT* gene promoter of cancer tissue in NSCLC patients was much higher than that in normal lung tissue and plasma, which showed a close association between NSCLC cancer and *MGMT* gene promoter methylation.

肺癌是目前发病率最高的恶性肿瘤, 其死亡率在男性为第一位, 女性为第二位。根据新近的流行病学资料显示, 每年全球肺癌新发病例高达120万人, 死亡病例约为100万人^[1]^。肺癌已成为对人类生存构成极大威胁的恶性肿瘤之一。近年来研究发现抑癌基因启动子甲基化与肺癌的发生有关, 如*P16*基因、*MGMT*基因、*APC*基因等, 但由于各个研究样本量较小, 统计效能较低, 说服力不强, 因此本研究采用*meta*分析的方法对*MGMT*基因启动子甲基化与非小细胞肺癌(non-small cell lung cancer, NSCLC)的相关性进行了探讨。

## 1 材料与方法

### 1.1 文献检索

计算机检索Medline、EMBASE、CNKI及万方等数据库, 收集公开发表的涉及*MGMT*基因启动子甲基化与NSCLC关系的临床研究。检索语种为英语和汉语, 分别以“MGMT”、“lung cancer”、“lung carcinoma”、“non-small cell lung carcinoma”、“methylation”为主题词和自由词, 检索Medline和EMBASE英文数据库; 以“肺癌”“非小细胞肺癌”“肺肿瘤”“*MGMT*”基因, “甲基化”为关键词或题名检索CNKI和万方等中文数据库。

### 1.2 文献纳入标准

① 研究对象经病理学或细胞学诊断为NSCLC患者; ②*MGMT*基因启动子检测采用甲基化特异PCR方法; ③研究结果提供癌组织与对照正常组织、血清、支气管灌洗液的甲基化率。

### 1.3 数据提取

由两名研究人员分别对纳入的研究进行相关数据提取, 包括一般资料如文题、作者、期刊杂志、研究所在地区等; 数据构成:包括NSCLC患者癌组织、正常肺组织、血浆及支气管灌洗液中*MGMT*基因启动子甲基化的发生率及甲基化模式。

### 1.4 统计学方法

以NSCLC患者癌组织与对照正常组织*MGMT*基因启动子甲基化率的优势比(odds ratio, OR)为统计指标。首先进行统计学异质性检验, 无统计学异质性采用固定效应模型合并数据, 存在统计学异质性则采用随机效应模型进行数据分析。所有统计分析采用Stata 11.0软件完成, 双侧*P*≤0.05认为差异有统计学意义。

## 2 结果

### 2.1 检索结果

检索相关数据库, 最初检索到相关文献137篇, 其中包括126篇英文文献, 11篇中文文献。严格依据纳入和排除标准, 进一步仔细阅读摘要及全文后剔除122篇, 最终有13篇文献符合要求并纳入本*meta*分析(图 1)。13篇文献中中文文献3篇, 英文文献10篇, 原始研究均提供了较为完整的数据资料(表 1)。

**1:**
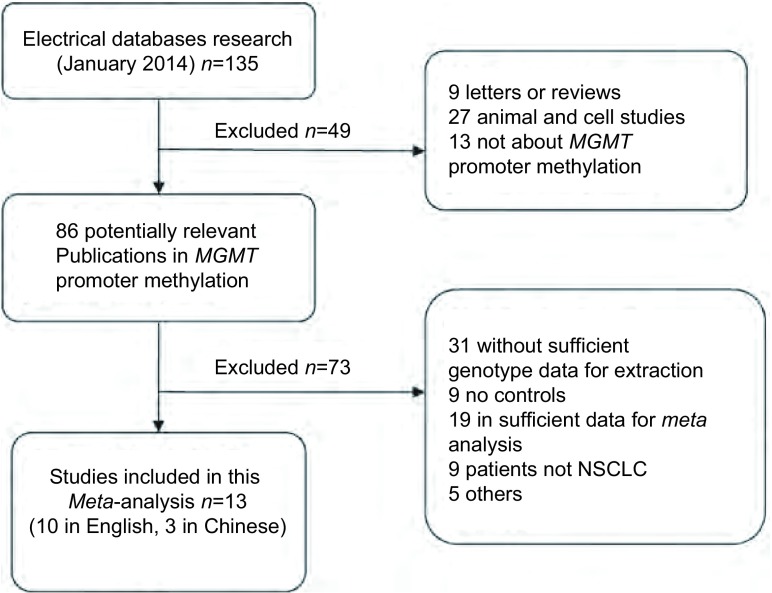
文献筛选流程图 Flow chart showing study selection procedure.NSCLC:non-small cell lung cancer

**1:** 纳入研究的基本特征 General characteristics of the included studies

Author	Tumor tissue (M+/M-)	Cotrol tissue (M+/M-)	Control type (Ⅰ/Ⅱ/Ⅲ/Ⅳ)	Method	Stage	Region	Time
Yao^[2]^	21/32	18/35	Plasm	MSP	-	China	2005
Zhang^[3]^	4/74	2/76	Tissue	MSP	25/33/19/1	China	2010
Kang^[4]^	19/34	15/38	Plasm	MSP	-	China	2011
Sabine^[5]^	22/85	0/104	Tissue	q-MSP	61/21/25/-	USA	2001
Brabender^[6]^	34/56	16/74	Tissue	MSP	44/19/27/-	Germany	2003
Guo^[7]^	14/6	8/12	Bronchial margins	MSP	-	China	2004
Russo^[8]^	18/15	6/27	Plasm	MSP	-	USA	2005
Topaloglu^[9]^	12/19	7/24	Bronchoalveolar fluid	MSP	17/9/5/-	USA	2004
Begum^[10]^	3/7	1/9	Plasm	q-MSP	-	USA	2011
Guo^[7]^	14/6	11/9	Bronchoalveolar fluid	MSP	-	China	2004
Guo^[7]^	14/6	1/13	Tissue	MSP	-	China	2004
Safar^[11]^	4/28	1/31	Tissue	MSP	-	USA	2005
Zhang^[12]^	4/74	2/76	Tissue	MSP	-	China	2011
Feng^[13]^	4/45	1/48	Tissue	MSP	21/17/-/-	USA	2008
Vallbohmer^[14]^	35/56	16/75	Tissue	MSP	45/19/27/-	USA	2006
MSP:Methylation-specific PCR; q-MSP:quantitative MSP.							

### 2.2 不同组织甲基化率比较

纳入本*meta*分析研究中NSCLC患者癌组织中甲基化的发生率为38%(95%CI:23%-53%); NSCLC患者正常肺组织、血浆和支气管灌洗液中*MGMT*基因启动子甲基化率分别为16%(95%CI:5%-27%)、23%(95%CI:10%-34%)和39%(95%CI:23%-55%)。

### 2.3 异质性检验

以肺癌中组织中*MGMT*基因启动子甲基化率的优势比OR为效应两, 进行统计学异质性检验。肺癌组织与血浆比较*I*^2^=41.4%, *P*=0.16;肺癌组织与正常肺组织比较*I*^2^=14.9%, *P*=0.37;肺癌组织与支气管灌洗液比较*I*^2^=0.0%, *P*=0.89, 均不存在统计学异质性, 统计分析均采用固定效应模型。

### 2.4 *MGMT*基因启动子甲基化与肺癌发生关系

以患者正常肺组织为参照, 肺癌中组织中*MGMT*基因启动子甲基化率的优势比OR=3.98(95%CI:2.71-5.84, *P* < 0.05);以患者血浆为参照, 肺癌中组织中*MGMT*基因启动子甲基化率的优势比OR=1.88(95%CI:1.16-3.05, *P* < 0.05);以患者支气管灌洗液为参照, 肺癌中组织中*MGMT*基因启动子甲基化率OR=2.05(95%CI:0.88-4.78, *P* > 0.05)(图 2)。

**2:**
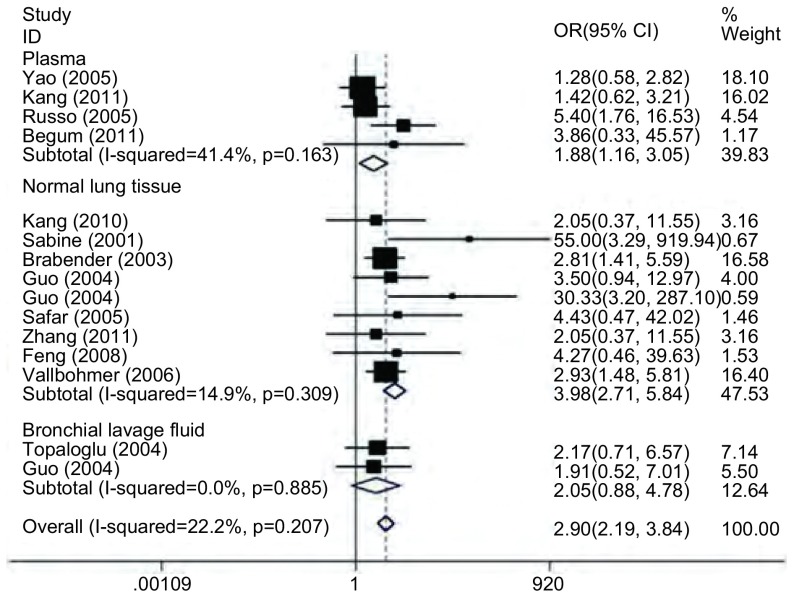
非小细胞肺癌患者癌组织与自身对照组织*MGMT*基因启动子甲率的森林图 Forest plot of the methylation rate in *MGMT* promoter between cancer tissue and autologous controls in non-small cell lung cancer (NSCLC) patients

### 2.5 敏感性分析

将纳入研究的每一篇文献逐一进行踢出, 评估单一研究对本*meta*分析结果的影响, 结果显示OR值置信区间始终变动范围在1.53-2.81之间, 始终大于1。因此, 本*meta*分析对纳入研究中的单一数据不敏感, 结论性质稳定。

### 2.6 发表偏倚的评估

根据*Egger*线性回归法及*Begg*漏斗图法(图 3)评估发表偏倚(*t*=1.55, *P* > 0.05), 提示研究结果不存在发表偏倚。

**3:**
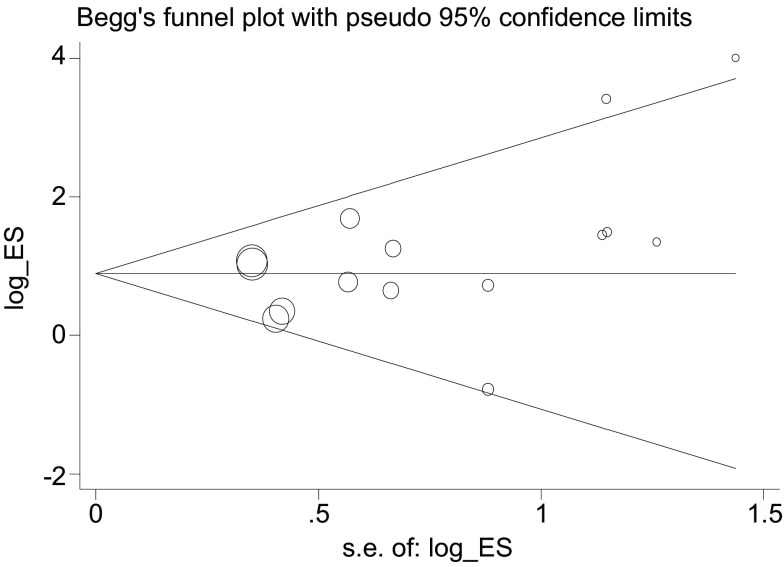
*Begg*漏斗图法评估发表偏倚 Publication bias evaluation by *Begg* funnel plot

## 3 讨论

肺癌为目前发病率最高的实体肿瘤, 每年全球新发病例高达120万, 严重威胁人类的健康。目前肺癌的确切发病原因并不完全清楚, 可能与遗传背景和后天因素有关。遗传背景包括性别、种族、家族肿瘤病史等, 后天已知肺癌发生的危险因素包括吸烟、环境污染、感染等。近年来研究发现抑癌基因的失活和癌基因的活化是肺癌发生的重要因素之一, 抑癌基因失活的一个重要原因为表观遗传修饰改变, 包括组蛋白甲基化、乙酰化、抑癌基因启动子甲基化和染色体重塑等^[11]^。其中抑癌基因启动子甲基化是目前研究的较为透彻的一种与肺癌发生有关的表观遗传修饰机制, 抑癌基因启动子区CpG岛中的胞嘧啶在甲基化转移酶(DNA methyltransferase, DNMT)的作用下发生甲基化, 从而抑制基因转录起始复合物与该基因的启动子区域结合, 导致该基因沉默, 继而导致抑癌基因表达下调, 促进肿瘤细胞的增殖分裂, 形成恶性转化。

*MGMT*基因编码产物为6-甲基鸟嘌呤DNA甲基转移酶, 该基因为DNA损伤修复基因, 对损伤的DNA可以进行修复, 进而阻止染色体的进一步损伤^[11]^。该基因全长为170 kDa, 包含有5个外显子, 编码一个由207个氨基酸残基组成的蛋白质, 该蛋白为DNA损伤修改的重要成分之一。已有研究显示^[7]^, 在多种肿瘤组织中*MGMT*基因的表达水平下调, 提示该基因的失活与多种肿瘤的发生有关。近年来的研究显示, 该基因的启动子区域CpG岛甲基化是导致该基因表达降低的一个重要原因。但各研究由于样本量较小, 研究结果存在一定的差异, 本研究采用*meta*分析的循证医学方法对*MGMT*基因启动子甲基化与NSCLC发生的关系进行了汇总分析, 研究结果显示与正常肺组织和血浆比较, 肺癌中*MGMT*基因启动子甲基化率增高(*P* < 0.05), 提示*MGMT*启动子甲基化与肺癌的发生可能存在相关性。

在本研究中, 我们对NSCLC患者癌组织和正常肺组织、血浆和支气管灌洗液中的*MGMT*基因启动子甲基化发生情况进行了循证医学分析, 结果提示*MGMT*基因启动子甲基化普遍存在于NSCLC患者癌组织中; 但在患者的正常肺组织和血浆中, 该基因的甲基化率较低, 说明癌细胞的转化与*MGMT*基因启动子甲基化密切相关, 该基因的失活在NSCLC的发生过程中可能起重要作用, 同时也提示甲基化转移酶抑制剂可能在治疗NSCLC当中扮演重要角色。
